# A novel whole-bacterial enzyme linked-immunosorbant assay to quantify *Chlamydia trachomatis* specific antibodies reveals distinct differences between systemic and genital compartments

**DOI:** 10.1371/journal.pone.0183101

**Published:** 2017-08-10

**Authors:** Hannah L. Albritton, Pamela A. Kozlowski, Rebecca A. Lillis, Chris L. McGowin, Julia D. Siren, Stephanie N. Taylor, Joyce A. Ibana, Lyndsey R. Buckner, Li Shen, Alison J. Quayle

**Affiliations:** 1 Department of Microbiology, Immunology, and Parasitology, Louisiana State University Health Sciences Center, New Orleans, LA, United States of America; 2 Department of Medicine, Division of Infectious Diseases, Louisiana State University Health Sciences Center, New Orleans, LA, United States of America; 3 Institute of Biology, University of the Philippines Diliman, Quezon City, National Capital Region, Philippines; University of California, San Francisco, Universit of California, Berkeley and the Childrens Hospital Oakland Research Institute, UNITED STATES

## Abstract

*Chlamydia trachomatis* (CT) is the leading sexually transmitted bacterial infection. The continued global burden of CT infection strongly predicates the need for a vaccine to supplement current chlamydial control programs. The correlates of protection against CT are currently unknown, but they must be carefully defined to guide vaccine design. The localized nature of chlamydial infection in columnar epithelial cells of the genital tract necessitates investigation of immunity at the site of infection. The purpose of this study was to develop a sensitive whole bacterial enzyme-linked immunosorbent assay (ELISA) to quantify and compare CT-specific IgG and IgA in sera and genital secretions from CT-infected women. To achieve this, elementary bodies (EBs) from two of the most common genital serovars (D and E) were attached to poly-L-lysine-coated microtiter plates with glutaraldehyde. EB attachment and integrity were verified by the presence of outer membrane antigens and the absence of bacterial cytoplasmic antigens. EB-specific IgG and IgA standards were developed by pooling sera with high titers of CT-specific antibodies from infected women. Serum, endocervical and vaginal secretions, and endocervical cytobrush specimens from CT-infected women were used to quantify CT-specific IgG and IgA which were then normalized to total IgG and IgA, respectively. Analyses of paired serum and genital samples revealed significantly higher proportions of EB-specific antibodies in genital secretions compared to sera. Cervical and vaginal secretions and cytobrush specimens had similar proportions of EB-specific antibodies, suggesting any one of these genital sampling techniques could be used to quantify CT-specific antibodies when appropriate normalization methodologies are implemented. Overall, these results illustrate the need to investigate genital tract CT antibody responses, and our assay provides a useful quantitative tool to assess natural immunity in defined clinical groups and CT vaccine trials.

## Introduction

*Chlamydia trachomatis* (CT) serovars D through K are obligate intracellular bacteria that reside in the human genital tract and are the most common bacterial sexually transmitted infection (STI) worldwide [1]. The endocervix is the primary site of infection in women, however CT can ascend into the upper genital tract and may cause pelvic inflammatory disease (PID) and infertility [2–6]. While the initiation of screening and antibiotic treatment programs has decreased these severe reproductive sequelae in some countries, including the US, rates of infection still continue to increase [7–9]. In addition, there is an estimated global prevalence of >100 million cases of CT, but approximately two-thirds of the world’s population has limited access to CT screening and treatment programs. The predominantly asymptomatic nature of CT infection also results in many undiagnosed individuals who go untreated, and hence, continue to spread infection. These untreated infections could also have other devastating consequences, as CT infection can also increase susceptibility to, and transmission of, human immunodeficiency virus (HIV) [10, 11]. All of these factors indicate the necessity for a CT vaccine to control the rise in infection incidence.

CT has a unique, generally biphasic, developmental cycle. Infectious, non-replicative elementary bodies (EBs) infect genital columnar epithelial cells and reside within a membrane-bound vacuole termed an inclusion. Here, EBs differentiate into non-infectious reticulate bodies (RBs), undergo replication, differentiate back into EBs, and exit cells via lysis or extrusion mechanisms [2, 12]. EBs released into the mucosal lumen can then infect nearby epithelial cells or be transmitted to sexual partners via genital secretions.

Due to the obligate intracellular nature of this bacterium and the complex interplay between the host and pathogen, CT can either evade or stimulate host immune responses, resulting in varying degrees of pathology and/or infection resolution [13–15]. Natural history studies indicate that untreated human infections typically persist for months to years if not treated [3, 16], and protection from re-infection is generally short-lived [17]. Interestingly, however, recent epidemiological studies by Geisler and colleagues identified a subset of women who naturally cleared their CT infection between diagnosis and treatment visits and re-infection rates in these women were significantly lower than in women with persisting infection. [18, 19]. These studies reveal a potentially useful cohort for identifying the correlates of immune protection against CT, which are imperative to define in order to guide CT vaccine design.

Our current understanding of the immune responses that may prevent CT infection is largely based on results from animal studies. These have indicated that both cellular and humoral immunity may play a role in bacterial clearance and protection from re-infection [20–25]. Experiments using various gene-knockout mice have indicated that IFNγ-secreting CD4 T cells predominantly clear primary CT infection [26–29]. However, both CD4 T cells and B cells are important to confer protection against re-infection [20, 21, 23–25, 30]. The mechanisms of how antibodies protect against CT infection are not well understood. Some studies have reported that CT-specific antibodies may play a role in neutralization, antigen-presentation, and T cell immunity [21, 23, 30]. Further, several studies have investigated titers of CT-specific antibodies in serum with results suggesting high levels of CT-specific IgG1 and IgG3 are indicative of current or recent CT infection [31, 32]. Taken together, these studies have suggested that an effective chlamydial vaccine may require induction of CT-specific antibodies, which warrants the characterization of humoral responses in natural chlamydial infections as well as clinical CT vaccine trials.

The two major antibody isotypes in genital secretions that may play a role in preventing CT transmission in humans are IgG, the predominant isotype, and polymeric secretory IgA (S-IgA), the main form of IgA in secretions [33, 34]. The majority of IgA and a significant proportion of IgG in human female genital secretions are derived from local plasma cells in the upper FGT [35]. Transport of IgG and IgA into FGT secretions is mediated by the neonatal Fc receptor (FcRn) and the polymeric immunoglobulin receptor (pIgR) respectively, both of which are expressed by columnar epithelial cells lining the endocervix and uterus [36–38]. Both of these antibody isotypes could function to prevent CT infection in the FGT by 1) In the lumen, IgG and S-IgA could bind to extracellular CT and block entry into the host cell by entrapping the bacteria in mucus [39, 40], 2) IgG and S-IgA could neutralize intracellular pathogens within columnar epithelial cells during transport via their respective receptors [41–44], 3) IgG and S-IgA could mediate protection or control of CT infection in the FGT mucosa via Fc-dependent functions, such as phagocytosis or enhanced antigen presentation, 4) IgG and IgA could mediate distinct functions in the FGT against CT and 5) Anti-CT IgG antibodies could mediate complement activation during infection [20, 45, 46], but IgA cannot. However, IgA may be more important in control of CT infection. A classic study by Brunham *et al*. demonstrated that the presence of CT-specific IgA, but not IgG, in cervicovaginal secretions of infected women inversely correlated with bacterial burden [47]. They also did not observe any correlation between bacterial burden and the presence of CT-specific antibodies in serum [47]. This study suggests that CT vaccines should induce local IgA responses in the FGT.

In vaccine trials, accurate measurement of CT-specific humoral responses in the genital tract milieu will require standardized and reproducible assays to determine if antibody responses correlate with protection. Here, we present a new whole-EB ELISA for quantifying IgG and IgA antibodies in sera and genital secretions against CT serovars D and E, two of the most common serovars in the US, comprising approximately 50% of infections in women [48–51]. We also describe sample collection and extraction methodology appropriate for measuring antibodies in human cervical and vaginal secretions. Utilizing these techniques and appropriate normalization methods, we determined that genital secretions from CT-infected women contain significantly greater proportions of EB-specific IgG and IgA when compared to serum. These results confirm the need to measure local immune responses when investigating the potential correlates of immune protection against CT. Our novel methodology provides a means to determine the concentration of EB-specific IgG and IgA at the local site of infection and directly compare them with those found in serum. This tool would be valuable in determining the threshold of local CT-specific antibody and antibody isotypes required for protection against CT infection, and thus will inform the design of an effective chlamydial vaccine.

## Materials and methods

### Preparation of CT EB stocks

Monolayers of mouse fibroblast L929 cells (obtained from Sigma Cat: 85011425) were grown to 95% confluency in Dulbecco’s Modified Eagle’s Medium (DMEM) supplemented with 10% fetal bovine serum (FBS) and L-glutamine. Cells were infected with purified CT EBs from serovar D (D/UW3/Cx) or E (E/UW5/Cx), resulting in infection rates of ≥ 95% [52]. Serovar D- and E-infected cells were harvested at 70 h post-infection. Chlamydial particles were released from cell lysates and supernatants by vortexing with 3 mm sterile glass beads (Fisher Scientific) for 30 seconds, placing on ice for 30 seconds, and then repeated the procedure 3 times. EBs were purified using a discontinuous Optiprep gradient centrifugation, as previously described, and confirmed to be infectious [53].

To determine the total protein concentration of purified EBs, 100 μl of each serovar preparation was first centrifuged at 13,000 g at 4˚C for 30 minutes. The pellet was then resuspended in 10 μl of sucrose-phosphate-glutamine buffer and treated with RIPA lysis buffer (ThermoFisher) supplemented with 1 μM phenylmethylsulfonyl fluoride and 20 μM dithiothreitol overnight at 4˚C. The following day, the total protein in the lysed preparation was measured using the bicinchoninic acid (BCA) assay according to the manufacturer’s instructions (Pierce). Each serovar preparation was subsequently diluted in SPG buffer such that they each contained equivalent levels of protein (362 μg/ml). Serovars were pooled in equal volumes and aliquots were stored at -8°C. Separate aliquots of each individual serovar were also made and stored under the same conditions.

### Antibodies

The following mouse monoclonal antibodies (mAbs) specific for CT outer membrane antigens were used: mouse anti-chlamydial lipopolysaccharide (LPS) IgG2b clone F1A6 [54], mouse anti-major outer membrane protein (MOMP) serovar E IgG3 clone CIVB5 (provided by Dr. You-xun Zhang, Boston University), mouse anti-MOMP serovar D IgG3 [55] (provided by Dr. Harlan Caldwell, NIAID/DIR), and mouse anti-cysteine-rich outer membrane protein A (OmcA) IgG2b [56] (provided by Dr. Guangming Zhong, University of Texas Health Sciences Center, San Antonio). For CT cytoplasmic antigens, the following antibodies were used: rabbit anti-Euo polyclonal antibody [57] (provided by Dr. Tom Hatch, University of Tennessee Health Sciences Center), mouse anti-heat shock protein 60 (Hsp60) IgG3 (provided by Dr. Y. Zhang) [58], and rabbit polyclonal anti-Scc4 protein [59] (provided by Dr. G. Zhong). Rabbit IgG (Life Technologies) and purified mouse IgG2b and IgG3 proteins (SouthernBiotech) were used as isotype-matched negative controls. In assays with human specimens, 2 IgG myeloma proteins (subclass unknown) and 9 IgA (4 IgA1 and 5 IgA2) myeloma antibodies (provided by Dr. Jiri Mestecky, University of Alabama at Birmingham) were utilized as negative controls. Other negative controls were the following: anti-HIV-1 gp120 and gp41 human IgG1 mAbs derived from EBV-transformed human B cells or hybridomas; 22.B and 7B2 [60] (provided by Dr. James E. Robinson, Tulane Medical School), 2F5 [61] (provided by Dr. Hermann Katinger, Polymun Scientific GmbH), F425 A1g8 [62], A32 [63], F105 [64] and 5F3 [61] from the NIH AIDS Reagent Program were utilized as additional negative controls. Additional negative controls were the 293T-expressed recombinant anti-Lassa virus 23.1D human IgG1 mAb [65] (provided by Dr. Robinson) and F425 A1g8 human IgA1 mAb [66] (provided by Dr. Lisa Cavacini, University of Massachusetts Medical School.

### ELISA for total IgG and IgA concentrations in specimens

Concentrations of total IgG and IgA were measured using a previously described ELISA [67–69]. Briefly, each well of a high-protein binding plate (Fisherbrand) was coated overnight at 4°C with either 0.25 ng of affinity-purified goat anti-human IgG γ chain or 0.5 ng IgA α chain-specific antibodies (ICN reagents; MP Biomedicals) in phosphate-buffered saline (PBS). Plates were washed with PBS containing 0.05% Tween in PBS (PBST), blocked with 2% goat serum (GS) (Equitech-Bio Inc) in PBST for 45 minutes at room temperature, loaded serial dilutions of test samples and previously calibrated IgG or IgA serum reference standards [67] and allowed to react overnight at 4°C. Plates were then washed 4 times and treated with 10 ng per well of biotinylated affinity-purified goat anti-human IgG γ chain or IgA α πϴchain-specific antibodies (Southern Biotech) for 1 h at 37°C. Plates were washed 4 times and reacted with 25 ng per well of neutralite avidin-labeled horse radish peroxidase (Southern Biotech) for 30 minutes at room temperature. After washing, the plates were developed with tetramethylbenzidine (Southern Biotech) for 20 minutes at room temperature. Absorbance was recorded at 370 nm in a SpectraMax plate reader (Molecular Devices, Sunnyvale, CA). The concentration of immunoglobulin in test samples was then interpolated from a 4-parameter standard curve constructed with the SoftMaxPro computer program (Molecular Devices).

### Optimized whole-EB ELISA

High-protein binding microtiter plates were coated overnight at 4°C with 75 μl per well of 1 μg/ml poly-L-lysine (Sigma) diluted in 0.05 M bicarbonate buffer, pH 9.6. Plates were then washed 3 times with 400 μl per well of PBST. A freshly thawed aliquot of pooled serovars D and E EBs was diluted in PBS to a final concentration of 4.5 μg/ml and 50 μl was added to each well. The plate was then centrifuged at 900 g for 5 minutes at room temperature. The EBs were fixed by adding 50 μl of 0.1% glutaraldehyde (Electron Microscopy Sciences) in PBS (for a final concentration of 0.05%) to all wells for 20 minutes at room temperature. The plates were then washed 3 times with PBST and blocked for 45 minutes at room temperature with 400 μL of 2% GS-PBST per well. After removing the blocking buffer, the wells were loaded with 100 μl of 2-fold serial dilutions of standards, 100 μl of 3 fold serial dilutions of test samples, and a positive control serum (from an infected subject), all diluted in GS-PBST. The IgG and IgA standards consisted of pooled human serum from CT-infected subjects that had been calibrated as described below. After an overnight reaction at 4°C, the plates were developed as described above. Absorbance was recorded at 450 nm after addition of 100 μl per well of 0.5M H_2_SO_4_. Concentrations of anti-EB IgG or IgA antibody in each sample were determined from standard curves, then divided by the concentration of total IgG or IgA to obtain the specific activity. The cut-off for positivity was considered to be the mean specific activity + 3 standard deviations (SD) determined by negative controls.

As in other studies [70], the concentration of specific antibodies in the EB-specific IgG or IgA standard were estimated by coating a plate with either anti-human IgG or IgA and adding the total IgG or IgA reference standards in duplicate to this portion of the plate. The remainder of the plate was treated with poly-L-lysine, EBs, and the EB-specific IgG or IgA standards. After the plate was developed, concentrations of antibodies in the EB-specific IgG and IgA standards were determined from standard curves constructed from the total IgG and IgA standards using a 4 parameter logistic curve fit model. This model takes into account maximum and minimum values that can be obtained, in addition to background absorbance being subtracted before standard calculation Further, concentrations for all samples were determined using the linear portion of the standard curve as absorbance values below and above the linear portion are less accurate.

In experiments analyzing the CT molecules present on plates, poly-L-lysine-coated plates fixed with whole EBs or an equivalent amount of lysed EBs were stained with mouse anti-CT IgG mAbs or rabbit polyclonal antibodies, which were then detected using biotinylated goat anti-mouse IgG (γ chain) or anti-rabbit IgG (H+L) antibodies (Southern Biotech). For the assays with lysed EBs, the EBs were lysed by adding Triton X-100 (final 0.1% v/v) to an EB aliquot, sonicating for 10 minutes, and allowing lysis to occur at 4°C for 72 h. The solution was then diluted to 10.5 ml in PBS and added at 100 μl per well to plates.

### Study population

Women aged 18 and above were recruited from the LSU CrescentCare Sexual Health Center in New Orleans, LA. Women were eligible for the study if they were returning to the clinic for treatment and counseling after a recent positive CT GENPROBE® APTIMA® nucleic acid amplification test (NAAT) from a cervical swab or urine sample. Individuals were excluded if they had cleared their CT infection between their screening and treatment visits. Other exclusion criteria included azithromycin and/or doxycycline treatment 8 weeks prior to their NAAT CT test, pregnancy, or HIV infection. Written informed consent was obtained from each woman, after which a standardized clinical questionnaire was completed. Matched serum, cervical and vaginal secretions, and cytobrush samples from 12 women were utilized in the present study. The median age of study participants was 23 (range 19–32); 66.7% were African American, 16.7% were white, and 8.3% white/Native American with the remainder not providing ethnicity information. All women were CT+ by a GENPROBE® APTIMA® NAAT assay from an endocervical swab on the day of enrollment. Median IFU was 4,671 (range 73–15,735) [71] and the CT *ompA*-based genotype was determined using an endocervical swab using previously described PCR methodology [72]. Genotypes from these specimens were as follows: E (16.7%), G (16.7%), Ia (33.3%), and F (8.3%). Three enrollees were unable to be genotyped. Fifty percent of women had a self-reported history of CT infection within the last 5 years. None of the enrollees were co-infected with *Neisseria gonorrhoeae*, and one enrollee was co-infected with *Trichomonas vaginalis*. *N*. *gonorrhoeae* infections were tested for by the presence of rRNA using GENPROBE® APTIMA® as instructed by the manufacturer, while *T*. *vaginalis* infection was determined by wet mount and real time PCR [73, 74]. Fifty percent of the recruited women had bacterial vaginosis, defined by a Nugent score of 7 or higher [75]. Mucopurulent cervicitis was observed in 33.3% of women upon pelvic examination. Of the enrollees, 16.7% (2/12) were on oral hormonal contraceptives (Orthotricyclen) and 8.3% (1/12) had levonorgestrel intrauterine systems (Mirena) in place. A total of 24 archived serum samples collected from a similar demographic cohort of women enrolled under identical inclusion/exclusion criteria at the Delgado Personal Health Clinic in New Orleans were also utilized in the current study [52, 71]. The studies and procedures for both patient cohorts were approved by the Louisiana State University Health Sciences Center Institutional Review Board.

### Collection and processing of specimens

Peripheral blood was drawn into SST Vacutainer™ blood collection tubes using standard phlebotomy, and serum was separated by centrifugation for 10 minutes at 425 g. Endocervical and vaginal secretions were collected with sterile Weck-Cel ophthalmic sponges (Beaver Visitec International) using methods similar to those previously described [76, 77]. Briefly, two sponges were consecutively placed in the cervical os to collect endocervical secretions, and 2 sponges were simultaneously placed in the posterior fornix of the vagina to collect vaginal secretions for 1 minute each. Sponges with absorbed secretions were placed in 5 ml cryovials and frozen at -80°C within 4 h of collection [67, 72, 77–79]. Genital secretions were eluted from sponges using spin assemblies as previously described [67]. Briefly, sponges were thawed and kept on ice during the elution process. One of the 2 cervical or vaginal sponges from each subject was placed in an upper chamber consisting of a 0.5 ml microcentrifuge tube with a hole punctured in the bottom. The upper chamber was placed inside a 2 ml cryovial that served as the lower chamber of the spin assembly. Three hundred μL of freshly prepared ice-cold elution buffer (0.25% of Igepal detergent and protease inhibitors in PBS) was added to the sponge. The spin assembly was then centrifuged at 20,000 g at 4°C for 1 h. The upper chamber with a dry sponge was then discarded and replaced with a new upper chamber containing the second cervical or vaginal sponge. Elution buffer was added and centrifugation was repeated. The lower chamber with the eluted cervical or vaginal secretion was then aliquoted and stored at -80°C.

A cervical cytobrush sample [71] was also collected by placing the cytobrush in the cervical os and gently rotating 360 degrees. The cytobrush was then immersed in 1.5 ml ice-cold transport medium containing keratinocyte serum free media (KSFM) with gentamycin and amphotericin B [71]. In order to process the sample, the cytobrush was briefly vortexed while still in transport media and was then pushed through a 1000 μL pre-cut pipette tip several times to collect mucus and cellular material that had adhered to the brush. The brush was discarded, and the medium was centrifuged for 10 minutes at 425 xg at 4°C to pellet cells. The supernatant was collected and frozen at -80°C and utilized to quantify antibody levels. Inclusion forming units (IFUs) were determined as previously described using a cervical swab placed in 1.5 ml of 0.2 M sucrose phosphate transport medium 16 for CT culture and genotyping [72].

### Statistics

All analyses were performed using GraphPad Prism software version 5.0 (GraphPad, La Jolla, CA) or SigmaPlot software version 11.2. The EB specific activity measured in serum of CT-infected women and negative controls was compared using the two-tailed Mann-Whitney rank sum test. Results for matched sera and secretions were compared using two-tailed Wilcoxon matched pairs rank sum tests. Only p values ≤ 0.05 were considered significant.

## Results

### Capture of whole EBs for the anti-EB ELISA

**Fig 1:**
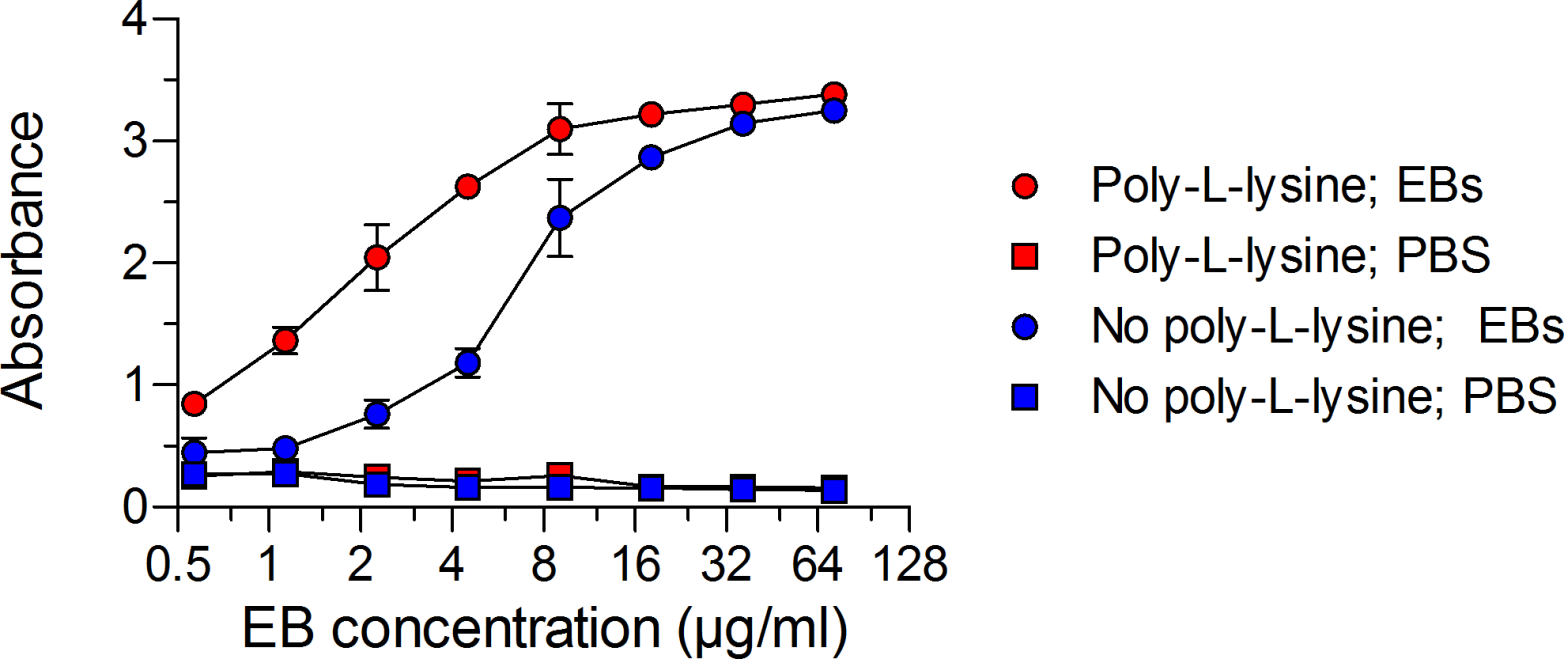
Poly-L-lysine coating of microtiter plates provides superior capture of EBs. Plates coated with poly-L-lysine or PBS were treated with serial dilutions of a pooled EB mixture (CT serovars D and E) in duplicate, centrifuged, and then fixed with glutaraldehyde. The plates were then washed, blocked, and incubated with a saturating concentration (5.3 μg/ml) of mouse anti-CT LPS mAb. Mean absorbance values ± SD from one representative experiment of three are presented. Poly-L-lysine or PBS-coated plates identically treated with PBS served as negative controls. The concentration of EBs shown represents the total protein in the pooled EB mixture. A concentration of 4.5 μg/ml of pooled EBs and lysed EBs was selected for use in subsequent experiments.

The first steps in the development of a CT-specific antibody ELISA were to (i) verify the efficient capture of whole EBs on the microtiter plate surface and (ii) ensure that the EBs were intact with only surface components exposed. Since both EBs and the high protein binding microtiter plates are negatively charged, we first determined whether poly-L-lysine, a positively charged synthetic polymer, would increase EB adherence to the plates. Serial dilutions of an EB mixture containing equal amounts of serovars D and E were added to uncoated or poly-L-lysine coated ELISA plates. The EBs were pelleted to the bottom of the plates by centrifugation and treated briefly with 0.05% glutaraldehyde. The optimal concentration of EBs of serovars D and E needed for detection of EB-specific antibodies was then determined by staining the plate with an anti-chlamydial LPS mAb. This mAb was chosen because (i) it recognizes LPS specific to the genus, thus encompassing all possible clinical CT strains, and (ii) LPS is a major outer surface component of EBs. As illustrated in Fig 1, poly-L-lysine coating of the plate resulted in superior EB capture compared to non-coated plates, and the optimal concentration of the EB stock was 4.5 μg/ml.

**Fig 2:**
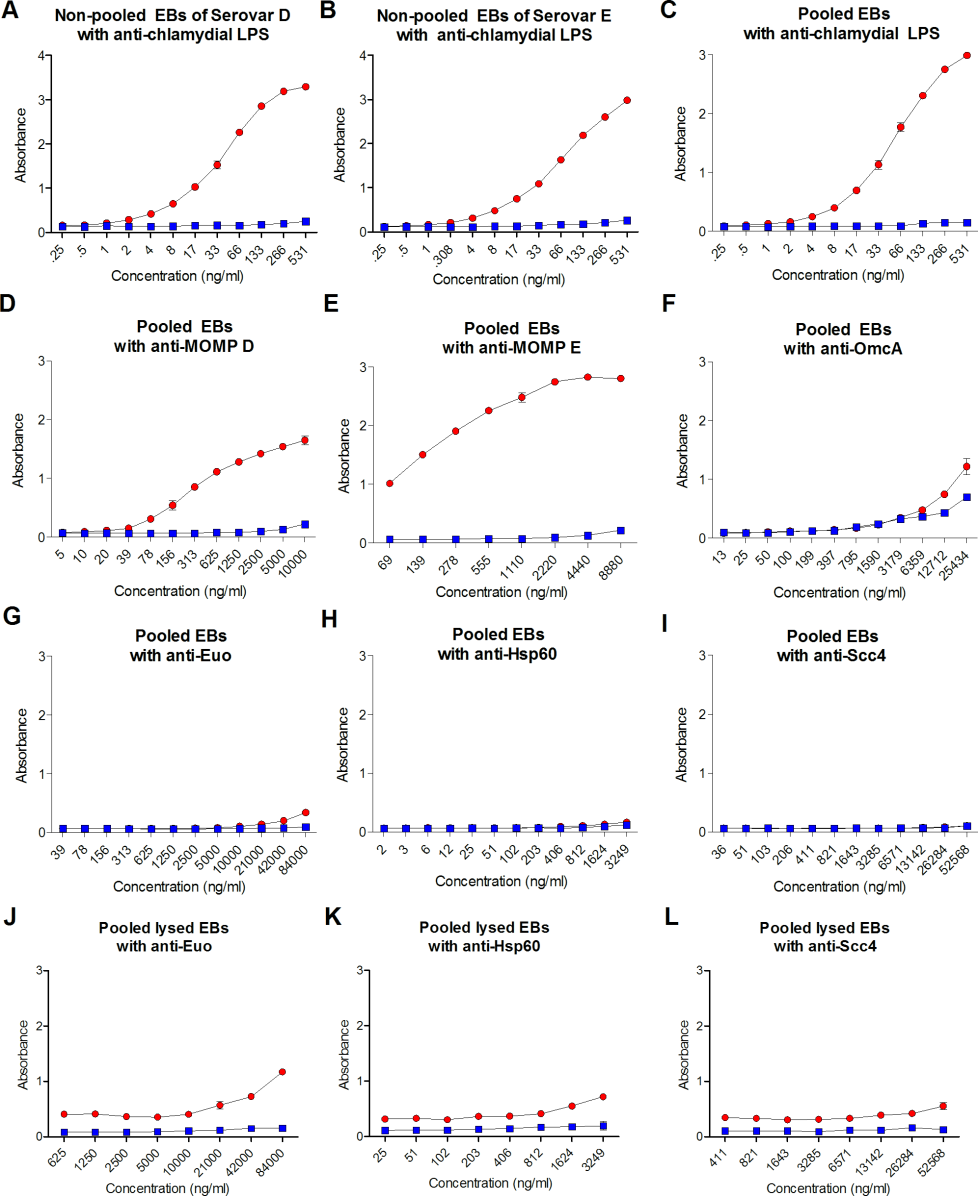
Chlamydial antigens measured on captured EBs. A total of 4.5 μg/ml of non-pooled or pooled EBs (red symbols) were fixed to poly-L-lysine-coated plates then incubated with duplicate serial dilutions of antibodies against chlamydial outer membrane proteins: **(A)** Non-pooled EBs of serovar D with mouse anti-CT LPS, **(B)** Non-pooled EBs of serovar E with mouse anti-CT LPS **(C)** Pooled EBs of serovars D and E with mouse anti-LPS **(D)** Pooled EBs of serovars D and E with mouse anti-MOMP D **(E)** Pooled EBs of serovars D and E with mouse anti-MOMP E and **(F)** Pooled EBs of serovars D and E with mouse anti-OmcA. Antibodies against cytoplasmic antigens of pooled EBs of serovars D and E: **(G)** Rabbit anti-Euo, **(H)** Mouse anti-Hsp60, and **(I)** Rabbit anti-Scc4. 4.5 μg/ml of lysed EBs of pooled serovars D and E were evaluated with **(J)** Rabbit anti-Euo **(K)** Mouse anti-Hsp60 and **(L)** Rabbit anti-Scc4. Blue symbols represent results obtained with irrelevant isotype-matched antibodies. Mean absorbance values ± SD from one representative experiment of three are shown.

Using the optimal EB concentration, we then verified that (i) serovars D and E EBs were present in the EB stocks and (ii) glutaraldehyde fixation of EBs did not impede the detection of other extracellular molecules. Using an anti-LPS antibody, EBs in both D and E non-pooled stocks (Fig 2A and 2B) in addition to pooled stocks (Fig 2C) were detected. MOMP for serovars D and E could be readily detected using mAbs (Fig 2D and 2E) in pooled stocks. A representative periplasmic protein, OmcA, which is normally cross-linked with MOMP in EBs, was detected using mouse anti-OmcA antibodies (Fig 2F). To confirm that whole EBs, not lysed EBs, were present on the plate, the cytoplasmic proteins Euo (Fig 2G), Hsp60 (Fig 2H), and Scc4 (Fig 2I) were also measured. Absorbance values for Euo, Hsp60, and CT protein Scc4 did not reach values above irrelevant antibody negative controls, indicating that these proteins remained intracellular after fixation of EBs. Lysed EBs were also added to poly-L-lysine coated plates and similarly fixed to confirm that the anti-Euo, Hsp60, and CT protein Scc4 antibodies could detect these antigens if they were present in whole EB ELISAs (Fig 2J–2L). Taken together these data confirm that (i) poly-L-lysine coating of microtiter plates results in the superior capture of EBs, (ii) major extracellular antigens can be detected using glutaraldehyde-fixed EBs, and (iii) EBs attached to plates maintain their structural integrity.

### Development of anti-EB IgG and IgA standards

**Fig 3:**
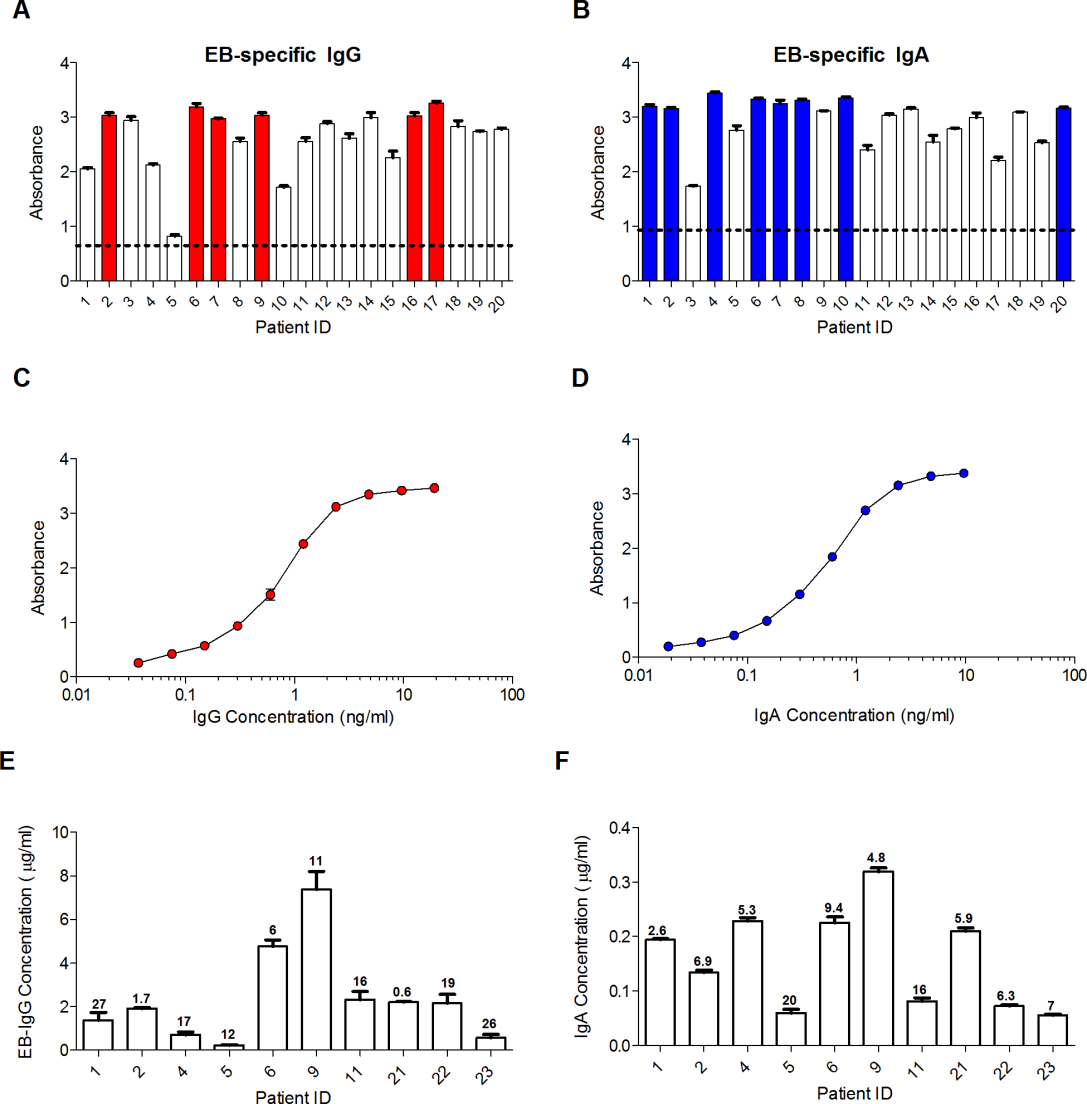
Creation of EB IgG and IgA standards for antibody quantitation. Sera from 20 CT-infected women were screened at a 1/1,000 dilution for **(A)** IgG antibodies and at a 1/50 dilution for **(B)** IgA antibodies that bound to pooled EBs. Shown is the mean absorbance ± SD for duplicate samples. The colored columns indicate sera that were subsequently pooled for use as the EB IgG or IgA standard and calibrated relative to total IgG and IgA standards, as described in the Methods. The dashed line represents the mean absorbance + 3 SD obtained with negative control IgG proteins at 10–20 μg/ml or IgA proteins at 20–40 μg/ml, concentrations approximately equivalent to those that would be present in serum diluted 1/1,000 (for IgG) or 1/50 (for IgA). **(C)** The EB IgG standard curve obtained after pooling the indicated sera is shown as mean ± SD for duplicate 2-fold serial dilutions, starting at a 1/1,000 dilution and a concentration of 19 ng/ml. **(D)** The EB IgA standard curve is similarly shown as mean ± SD for duplicate 2-fold dilutions starting at a 1/50 dilution and a concentration of 9.6 ng/ml. Using the EB IgG and IgA standards, **(E)** anti-EB IgG and **(F)** anti-EB IgA antibodies were measured in serum from 10 CT-infected women. Shown is the mean concentration ± SD obtained in two separate experiments. The inter-assay deviation for each sample is shown as the % CV (100% x SD/mean) above each column.

There are currently no anti-CT human IgG or IgA mAbs that could be used as standards in an ELISA for measuring EB-specific antibodies. We therefore utilized archived serum from CT-infected women to create standards for quantifying EB-specific IgG and IgA concentrations in specimens. Banked serum samples from 20 CT-infected women were screened for anti-EB antibodies. Six serum samples which consistently produced absorbance values > 2.9 at a 1/1,000 dilution were pooled for use as the EB IgG standard (Fig 3A). Eight serum samples which consistently produced absorbance values > 3 at a 1/50 dilution were pooled for use as the EB IgA standard (Fig 3B). The standard curves obtained with these pooled sera are illustrated in Fig 3C and 3D. The intra-assay deviation for samples assayed in duplicate was minimal, with coefficients of variation (CV) measuring at approximately < 1% (Fig 3C and 3D). Inter-assay reproducibility in ELISAs with the EB IgG and IgA standards was also found to be excellent, as demonstrated in Fig 3E and 3F using 10 randomly selected serum samples from CT-infected women. Concentrations of EB-specific antibodies measured in these sera on 2 different days did not differ by more than 1.7-fold. Indeed, the mean % CV for EB-specific IgG and IgA concentrations obtained with these samples was only 13.7% and 8.5%, respectively, which corresponded to 1.3- and 1.2-fold differences. Mean % CV should be 20% or less. These results demonstrate that results obtained in the EB assay are highly reproducible.

### EB-specific antibodies in serum of women with genital CT infection

**Fig 4:**
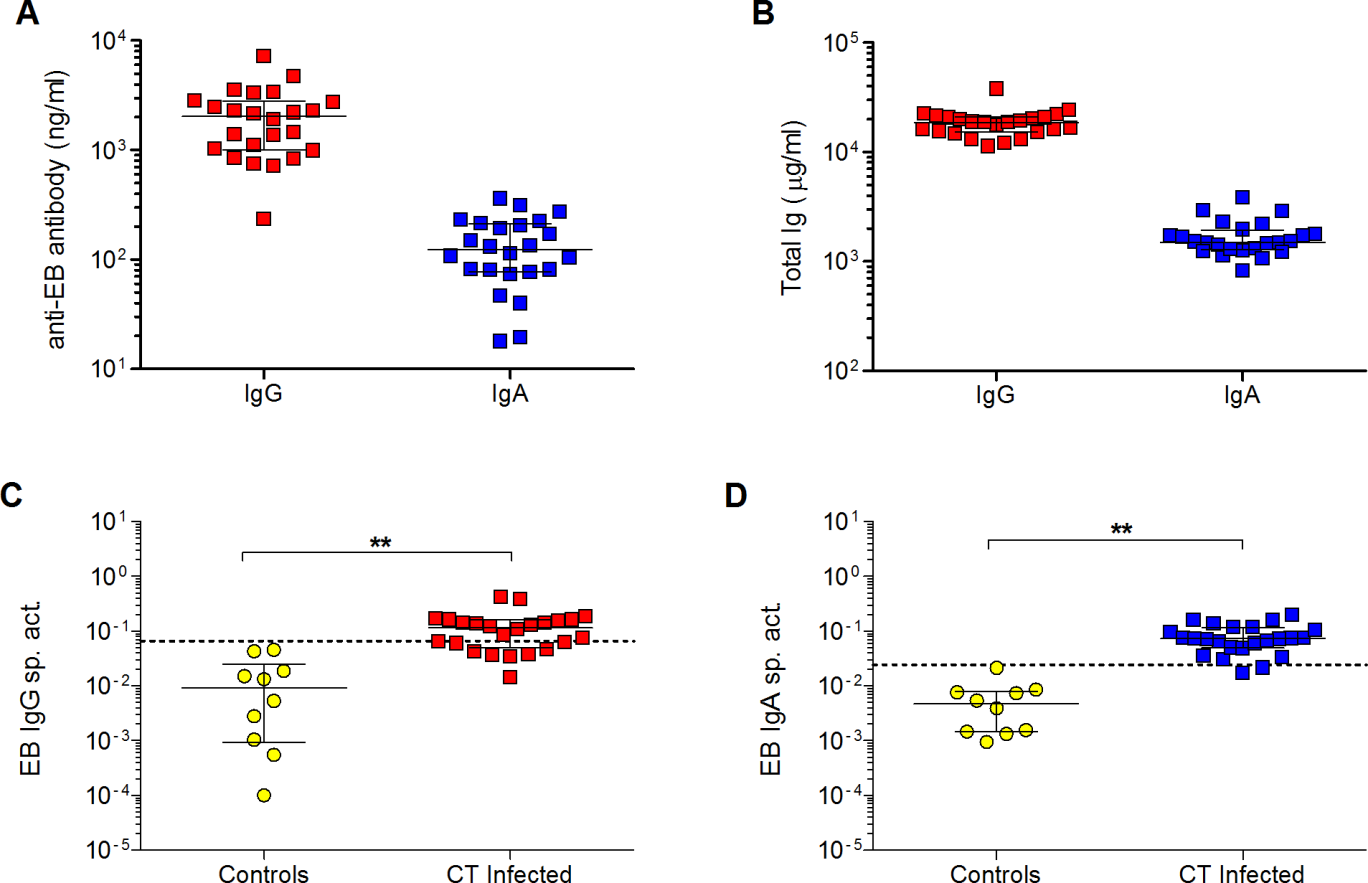
Quantification of EB-specific IgG and IgA in serum of CT-infected women. **(A)** Concentrations of anti-EB IgG or IgA antibodies were measured in serum from 24 CT-infected women using the EB IgG or IgA standard. **(B)** Concentrations of total IgG and IgA in the sera were also measured by ELISA. The magnitude of systemic antibody responses to CT in infected women was determined by calculating the **(C)** EB IgG or **(D)** IgA specific activity (ng anti-EB IgG or IgA antibody per μg total IgG or IgA, respectively). The thresholds for significance in each assay are indicated by dashed lines representing the mean specific activity + 3 SD for negative controls (IgG or IgA myeloma proteins or human monoclonal antibodies to irrelevant antigens). Bars in all graphs denote medians and interquartile ranges. The EB specific activity in CT-infected women and controls was compared using the two-tailed Mann-Whitney rank sum test. **p < 0.01.

Next, we used the EB IgG and IgA standards to quantify EB-specific IgG and IgA in archived serum samples from 24 women with current endocervical CT infections. As shown in Fig 4A, concentrations of anti-EB IgG in serum were higher than concentrations of anti-EB IgA antibodies. However, this was likely due to the fact that total IgG in serum is typically higher than total IgA (Fig 4B). As both IgG and IgA antibody levels can vary by age and ethnicity, the anti-EB IgG or IgA concentration in each serum sample was adjusted to the total IgG or IgA concentration to obtain the specific activity, and the magnitude of the systemic IgG and IgA response to CT was found to be comparable (Fig 4C and 4D). As it is difficult to obtain serum samples from human subjects who are known to have never been exposed to CT, we used 2 IgG myeloma proteins and 8 anti-HIV human IgG mAbs as negative controls to establish the threshold for significant specific activity in the EB IgG ELISA. Similarly, 9 IgA myeloma proteins and 1 anti-HIV human IgA1 mAb served as negative controls in the EB IgA assays. As illustrated in Fig 4C and 4D, 75% (18/24) and 96% (23/24) of CT positive patients had significant EB IgG and IgA specific activity, respectively. The 25% frequency of CT-infected women found in this study to be negative for CT-specific IgG antibodies in serum is similar to that reported for a cohort with similar demographics [18]. The lack of CT-specific serum IgG in some of these women could be related to the fact that CT infection is localized to the genital tract.

### EB-specific antibodies in serum and genital secretions

**Fig 5:**
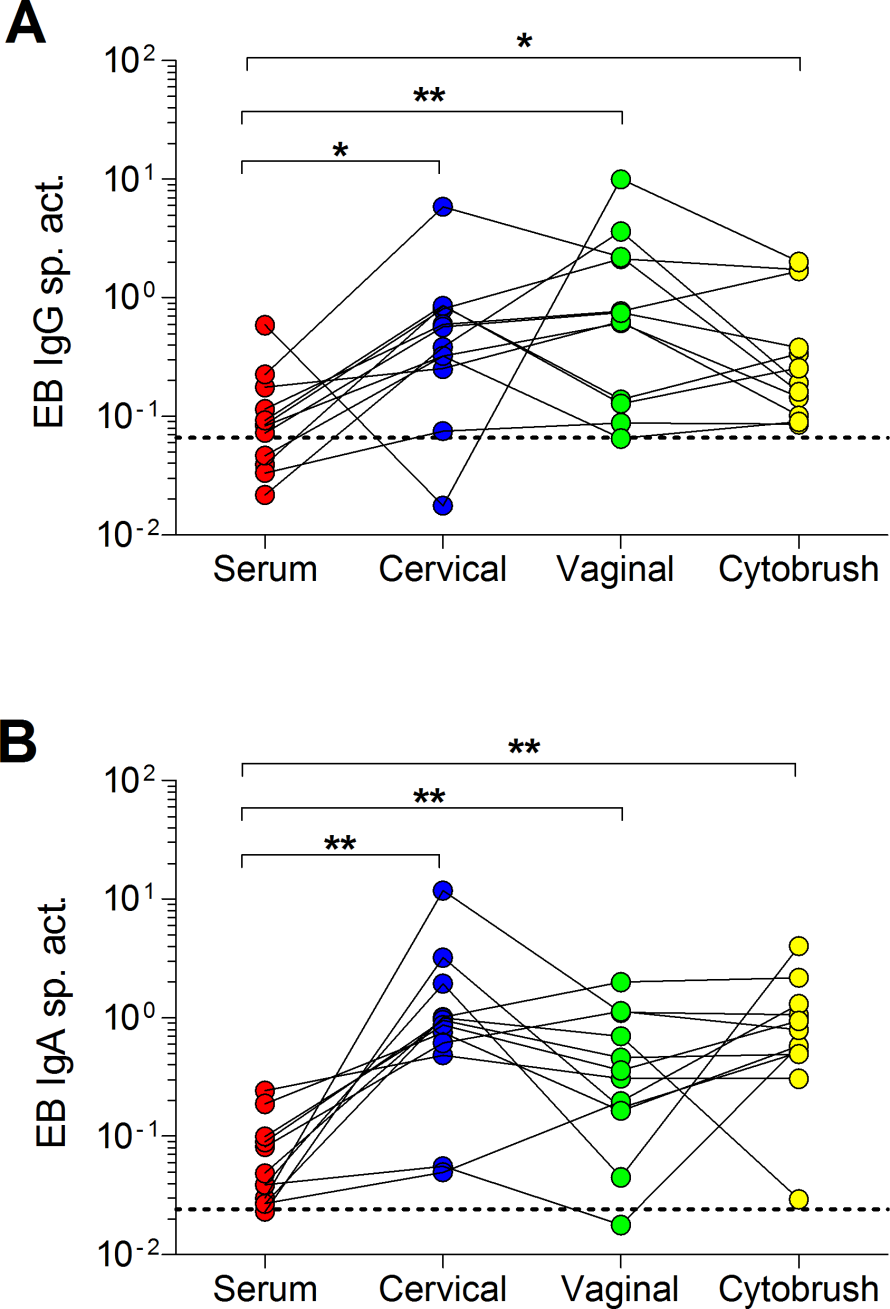
EB IgG and IgA specific activity in serum and genital tract secretions in CT-infected women. **(A)** EB IgG and **(B)** IgA specific activity in matched serum, endocervical and vaginal secretions, and cytobrush samples were determined by dividing the anti-EB IgG or IgA antibody (in ng/ml) by the respective total IgG or IgA concentration (in μg/ml) in each sample. Dashed lines denote the thresholds for significance (mean specific activity + 3 SD of negative controls). *p < 0.05 and **p < 0.01 by two-tailed Wilcoxon matched pairs rank sum test.

Finally, we compared EB-specific IgG and IgA in matched sera and genital secretions from 12 patients with a positive endocervical CT NAAT, indicative of a current infection (Table 1, Fig 5A and 5B). Because concentrations of total and specific antibodies in genital secretions fluctuate throughout the menstrual cycle [76, 80], and genital secretions were collected at different phases of the menstrual cycle, it was critical to determine the EB IgG and IgA specific activity by expressing results as the ratio of EB-specific antibody concentration to total antibody concentration. This ratio allows comparison of the magnitude of mucosal immune responses across patients and between matched sample types [76, 80]. Similar to results using the archived samples, serum from 67% (8/12) of these patients were positive for EB-specific IgG (Fig 5A). Enrollees that were negative for EB-specific IgG were infected with either genotype Ia, E, or F with one enrollee unable to be genotyped. In contrast, EB-specific IgG responses were detected in 92% (11/12) of endocervical and vaginal secretions and 100% of cytobrush supernatant samples. The enrollee that was negative for endocervical EB-specific IgG was infected with genotype Ia while the enrollee negative for vaginal EB-specific IgG was infected with F. For IgA, 92% (11/12) women had anti-EB IgA in serum, EB-specific IgA was detected in 92% (11/12) vaginal secretions, and 100% of cervical secretions and cytobrush supernatants (Fig 5B). The enrollee negative for serum EB-specific IgA was infected with genotype E as was the enrollee negative for vaginal EB-specific IgA. Importantly, cervical (p = 0.0122 for IgG and p = 0.0005 for IgA) and vaginal (p = 0.0005 for IgG and p-0.0049 for IgA) secretions and the cytobrush samples (p = 0.0161 for IgG and p = 0.001 for IgA) had significantly greater proportions of EB-specific IgG and IgA when compared to matched serum. However, among the genital specimens, there were no significant differences in proportions of EB-specific IgG or IgA (Fig 5A and 5B). These results reaffirm basic mucosal immunologic tenets which claim that blood is unlikely to be as sensitive as mucosal samples for assessing site-specific immune responses to localized mucosal pathogens [81–85]. There were no significant differences between proportions of EB-specific IgG and IgA among the genital samples, indicating that vaginal secretions or cytobrush supernatants may possibly be used instead of, and/or in addition to, cervical secretions during clinical studies.

**Table 1:** Total and EB-specific antibodies in serum and secretions of CT-infected women.

	Serum				Vaginal^a^				Endocervical^a^				Cytobrush^b^			
	Total	Total	EB	EB	Total	Total	EB	EB	Total	Total	EB	EB	Total	Total	EB	EB
	IgG	IgA	IgG	IgA	IgG	IgA	IgG	IgA	IgG	IgA	IgG	IgA	IgG	IgA	IgG	IgA
	(μg/ml)	(μg/ml)	(ng/ml)	(ng/ml)	(μg/ml)	(μg/ml)	(ng/ml)	(ng/ml)	(μg/ml)	(μg/ml)	(ng/ml)	(ng/ml)	(μg/ml)	(μg/ml)	(ng/ml)	(ng/ml)
	n = 12	n = 12	n = 12	n = 12	n = 12	n = 12	n = 12	n = 12	n = 12	n = 12	n = 12	n = 12	n = 12	n = 12	n = 12	n = 12
**Median**	14118	1264	1011	58	51	12	22	4	229	26	89	23	44	7	7	5
**Mean**	14103	1339	1750	98	123	17	656	11	235	36	110	57	73	11	81	18
**Maximum**	19755	2206	7311	309	712	22	7310	60	520	80	247	235	225	28	389	62
**Minimum**	8241	828	382	27	4	2	3	0	33	9	9	1	3	1	1	0

^*a*^Concentrations shown are for those measured in diluted (eluted) secretions collected with Weck-Cel sponges.

^*b*^Concentrations measured in medium from immersed cytobrushes.

*Note that EB-specific IgA and total IgA antibodies in secretions are likely primarily polymeric*, *and hence*, *they may be under-estimated by a factor of 2*.*5 as they were measured relative to a monomeric IgA serum standard* [70]

## Discussion

A safe and effective vaccine against CT could either create sterile immunity or generate an immune response that limits CT replication to the endocervix and prevents ascension to the upper genital tract. A recent clinical study identified a cohort of women who can naturally resolve their infection and were subsequently protected from re-infection, providing evidence that natural immunity against CT can generate some level of protective immunity [18]. It is likely that T cells contribute in immunity to CT [21, 86]. However, animal studies strongly support a role for antibodies in CT clearance upon secondary infection [20, 21, 23, 30, 87]. In CT-infected women, the presence of anti-CT IgA in genital secretions has also been inversely correlated with cervical bacterial burden [47]. Antibodies could function to prevent infection of epithelial cells and clearance of CT via 1) extracellular or intracellular neutralization, 2) Fc-mediated mechanisms such as antibody-dependent phagocytosis or IgG-mediated complement fixation, and/or 3) enhancement of T cell responses, such as IFNγ production, which are known to be dependent upon antibody isotype. [21, 23, 30, 41–44, 88]. However, the levels of anti-CT genital antibodies required for protection or clearance are not known and need to be elucidated in humans using quantitative assays. Antibody isotype may also be important for protection. For example, vaccine-induced anti-MOMP IgA in the genital tract of mice has been associated with reduced CT infection upon challenge [25], but anti-MOMP IgG has been associated with exacerbated pathology and delayed CT clearance [24]. Thus, it is imperative to investigate both the levels of IgG and IgA responses against CT.

Developing an effective CT vaccine and moving clinical trials forward will require technical tools to quantitate and identify antibodies that may be associated with protection against CT. We therefore created and optimized a robust ELISA for quantifying CT-specific antibodies using whole chlamydial EBs. We determined that poly-L-lysine significantly increased EB adherence to microtiter plates. This is most likely due to the positively-charged poly-L-lysine improving the electrostatic interactions between the negatively-charged microtiter plate and EBs; poly-L-lysine has also been shown to be more effective for coating plates with LPS when compared to other adherents [89, 90]. Glutaraldehyde also contributed to the robustness of this assay, as it successfully fixed EBs on the plate [91]. It was possible that fixation could have destroyed antibody epitopes on EB outer surface molecules. Therefore, we confirmed that the major outer surface components, LPS and MOMP, could be recognized on fixed EBs by antibodies. We also confirmed that the structural integrity of freeze-thawed EBs attached to the plates was maintained, as bacterial cytoplasmic proteins were not present in significant quantities. This is important because antibodies to outer membrane proteins of the infectious EB particles are most likely to prevent CT infection, and thus are the more desirable outcome measure in this ELISA.

Based on previous studies, we developed a clinical sampling protocol that included standard venipuncture to acquire serum, absorbent ophthalmic sponges to collect cervical and vaginal secretions, and cytobrush specimens [52, 71, 72, 77]. The advantage of these sponges is that they can be used to sample specific anatomic sites in the FGT, and the secretions eluted are less dilute than standard cervicovaginal lavages, which facilitates the detection of low levels of antibodies. Dilution factors introduced during the elution process can also be determined [67] if the absolute concentration of a specific antibody in the original secretion is desired for determining threshold concentrations for a vaccine candidate efficacy, for example. Both IgG and IgA antibody levels in genital secretions vary by age, ethnicity, and menstrual cycle phase [76, 77, 80]. To accurately compare the magnitude of antibody responses in the FGT of human subjects, it is optimal to determine the proportion of specific antibodies relative to the total concentration of IgA or IgG, which does not require a dilution factor be applied. While this method of normalization will not identify concentrations of CT-specific IgG and IgA that are associated with protection these levels can still be identified using this quantitative assay. Since vaginal sampling with sponges is less invasive and can provide a greater volume of secretion compared to the endocervix, we investigated if antibody levels in the posterior fornix of the vagina would reflect levels measured in the cervix. We found no significant differences in the proportions of CT-specific antibodies present in secretions collected from these two sites.

We also examined the usefulness of endocervical cytobrush supernatants [71, 92, 93] for quantification of antibodies in the FGT. We observed no significant differences in the proportions of CT-specific antibodies in cytobrush supernatants when compared to vaginal or cervical sponge eluates, suggesting that all three of the sampling techniques may be suitable for collection and quantification of CT-specific antibodies. However, the total IgA and IgG levels in cytobrush supernatants were considerably lower than those in vaginal and cervical secretions, which may hinder antibody detection in other studies. Immersing cytobrushes in a smaller volume of medium than that used in the current study might remedy this problem. Nevertheless, vaginal sponge secretions may be the preferable sample as women are more likely to enroll in studies when less invasive techniques are employed, including those obtained by self-collection [94]. In this study, we found that FGT secretions more often contained CT EB-specific IgG and IgA antibodies compared to serum. Importantly, when antibodies were detected in both secretions and serum, the proportions of CT-specific IgG and IgA antibodies in the secretions were significantly higher. These results clearly indicate that CT infection generates a greater antibody response at the site of infection. This is not surprising considering that the majority of serum antibodies are derived from plasma cells in the bone marrow [95], but CT infections are generally confined to columnar epithelial cells and rarely disseminate beyond the genital tract. It is noteworthy that CT infection generates a local IgG response in the FGT. Most of the IgG in FGT secretions has been presumed to be derived from serum IgG antibodies that passively diffuse from tissue between squamous epithelial cells in the lower tract (vagina/ectocervix) or that are actively transported from the serum by FcRn-expressing columnar epithelial cells in the endocervix and uterus. However, a greater number of the plasma cells in the cervical transformation zone of women have been found to produce IgG than IgA [35]. It is also possible that IgG antibodies in the FGT of CT-infected women may be derived from plasma cells in local ectopic lymphoid follicles, which have often been observed in the endocervix, transition zone, and ectocervix of CT-infected women [96, 97]. The significance of IgG and IgA antibodies locally produced in the mucosa in CT infection are currently unknown. Their combined efforts may be beneficial for preventing CT infection. Quantitation of EB-specific IgG and IgA responses in the genital tract of women and determining their role in CT clearance, pathology, and protection may aid in understanding the functions of locally produced antibodies.

While it is possible the enrollees that were negative for EB-specific antibodies could be due to the use of serovars they were not currently infected with, we did observe negativity with enrollees currently infected with E. Serovar specific immunity has been proposed as a mechanism of protection against re-infection of the same serovar [98, 99]. However, in a human trachoma infection, serovar cross-reactive antibodies were observed over time [100] and in the murine model, protection was observed after heterotypic challenge [101], suggesting serovar-cross reactive antibodies may be present and assist in protection against re-infection.

In summary, we have developed a whole EB ELISA for quantifying antibody responses to CT in both serum and genital secretions. Other EB serovars could likely be utilized in this assay to measure systemic and mucosal humoral responses to CT in vaccine trials. Importantly, we found significantly higher proportions of CT-specific IgG and IgA in all genital sample types when compared to matched serum. Future studies should explore how mucosal IgA or IgG antibodies to CT correlate with clearance of infection or with ascension of infection and upper tract pathology, such as PID. This knowledge could then be applied to recapitulate natural protective immunity in a vaccine, and our ELISA could be utilized in clinical trials to confirm that these protective humoral responses have been generated.
